# Neurofilament light chain predicts risk of recurrence in cerebral amyloid angiopathy-related intracerebral hemorrhage

**DOI:** 10.18632/aging.103927

**Published:** 2020-11-18

**Authors:** Xin Cheng, Ya Su, Qiong Wang, Feng Gao, Xiaofei Ye, Yiqing Wang, Yiwei Xia, Jiayu Fu, Yong Shen, Rustam Al-Shahi Salman, Qiang Dong

**Affiliations:** 1 Department of Neurology, National Clinical Research Centre for Aging and Medicine, Huashan Hospital, Fudan University, Shanghai, China; 2 Department of Neurology, First Affiliated Hospital of University of Science and Technology of China, Hefei, China; 3 Neurodegenerative Disorder Research Centre and Institute on Aging and Brain Disorders, University of Science and Technology of China, Hefei, China; 4 Department of Health Statistics, Second Military Medical University, Shanghai, China; 5 Centre for Excellence in Brain Science and Intelligence Technology, Chinese Academy of Sciences, Shanghai, China; 6 Centre for Clinical Brain Sciences, University of Edinburgh, Edinburgh, UK

**Keywords:** cerebral amyloid angiopathy, blood biomarkers, intracerebral hemorrhage, recurrence, neurofilament light chain

## Abstract

Predicting recurrent intracerebral hemorrhage (ICH) related to cerebral amyloid angiopathy (CAA) currently relies on brain images. We aimed to investigate whether blood neurodegenerative biomarkers predict disease severity and ICH recurrence in CAA. We recruited 68 first probable CAA-ICH cases from a Chinese prospective cohort, and 95 controls. We used the single-molecule array to measure acute phase blood amyloid-40, amyloid-42, total tau and neurofilament light chain (NfL). We used multivariable Cox regression models to assess the association between blood biomarkers and CAA-ICH recurrence, and used the concordance (c-) index to assess prediction models. Blood amyloid-42/40, total tau, and NfL levels changed in CAA-ICH cases than controls. During a median follow-up of 2.4 years, NfL was associated with CAA-ICH recurrence (adjusted hazard ratio 2.14, 95% CI 1.57-2.93) independent of MRI burden of small vessel disease (SVD). The performance of a model to predict CAA-ICH recurrence using MRI burden of SVD alone (c-index 0.77) increased with the addition of NfL (c-index 0.88, 95% CI 0.73-1.00, p=0.019). Further, NfL was associated with baseline ICH volume, NIHSS and 6-month mRS score. Blood NfL is associated with severity and prognosis of CAA-ICH and is a promising addition to MRI burden of SVD to predict CAA-ICH recurrence.

## INTRODUCTION

Cerebral amyloid angiopathy (CAA) is one of the leading causes for spontaneous lobar intracerebral hemorrhage (ICH) in the elderly. Patients with CAA show substantially higher risks of recurrent ICH at a rate of 6.9 - 10% per year [1–3] compared with deep ICH of about 3% per year [4], leading to subsequent disability and mortality. So far, the prediction of ICH recurrence and disease severity for CAA mostly depends on brain imaging features, including the number of previous ICH and cerebral microbleeds (CMBs), posterior white matter hyperintensities (WMH) [1, 5] and cortical superficial siderosis (cSS), among which cSS is most clinically relevant [2, 3]. However, CAA-related MRI features represent vascular endpoints of CAA rather than neurodegenerative processes that might influence disease progression and recurrence risk [6].

Growing evidence suggests the interplay between vascular damage and neurodegeneration in Alzheimer’s disease and CAA [7, 8]. Molecular biomarkers, especially blood-based, may provide an easy approach in directly measuring the underlying processes [9, 10]. However, the utilities of circulating biomarkers for CAA are still underexplored except for the diagnostic value of APOE genotype, β-amyloid (Aβ) 42 and Aβ40. APOE ε4 possession has strong association with CAA pathology and helps diagnose CAA-ICH [11], but may not be generally available worldwide. The decreased cerebrospinal fluid Aβ42, Aβ40 and total tau serve as biomarkers for distinguishing CAA from controls [6], while the diagnostic utilities of blood Aβ and tau are still controversial [12, 13]. As a structural scaffolding protein in the brain, neurofilament light chain (NfL) is highly specific for axonal injury and eventual neuronal cell death and shows prognostic values in various central nervous system diseases [14], which has not been well studied in CAA. Therefore, we hypothesized that neurodegenerative biomarkers might predict further vascular damage. We aimed to examine whether blood biomarkers of Aβ42, Aβ40, total tau and NfL in CAA indicate disease severity and predict prognosis.

## RESULTS

**Figure 1:**
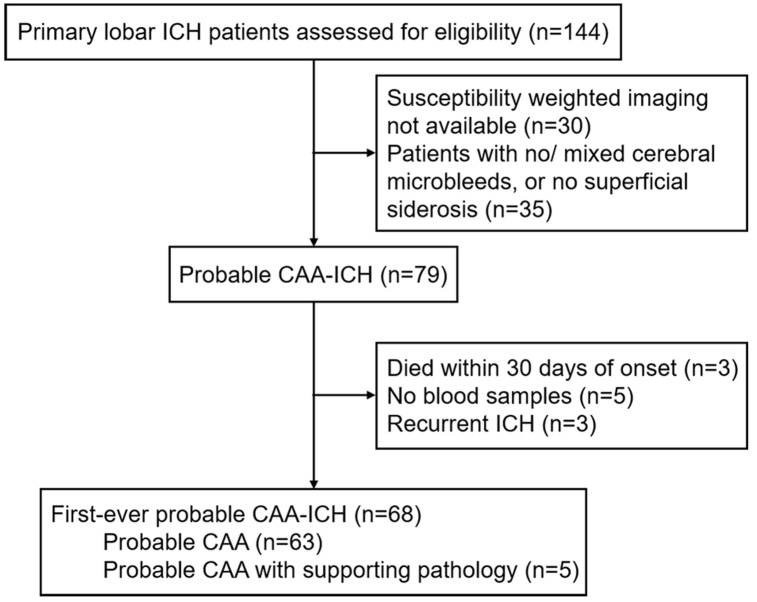
**Study flow chart.** Abbreviations: ICH, intracerebral hemorrhage; CAA, cerebral amyloid angiopathy.

A total of 144 cases with primary lobar ICH were admitted in two independent hospitals in China during the study period, and finally 68 cases with first-ever probable CAA-ICH (Figure 1) and 95 healthy elder controls were included in the study.

### Study population

The demographic data, clinical manifestations and imaging characteristics of healthy controls, and cases with CAA-ICH overall and according to lobar ICH recurrence were shown in Table 1. For cases with CAA, the mean ± standard deviation (SD) age was 70.1±9.1 years and 73.5% were males. During a median (interquartile range [IQR]) follow-up of 2.4 (1.3-4.0) years, 19 (27.9%) patients had recurrent lobar ICH without deep ICH. The ICH recurrence was associated with older age, higher degree of periventricular spaces in the centrum semiovale (CSO-PVS) and heavier total MRI burden of small vessel disease (SVD), but not lobar CMBs count, cSS presence or extent, or WMH Fazekas score in univariable Cox regression analyses in our cohort (Table 2).

**Table 1:** Demographic, clinical and imaging data and blood biomarkers of healthy controls and cases with probable CAA-ICH (subdivided according to ICH recurrence).

**Variables**	**Healthy controls**	**First-ever probable CAA-ICH**	***P* Value**	**CAA with lobar ICH recurrence**
	**(n=95)**	**(n=68)**		**No (n=49)**	**Yes (n=19)**
Age of onset, y	69.0 ± 8.3	70.1 ± 9.1	0.43	68.5 ± 9.5	74.1 ± 6.9
Sex, male	63 (66.3)	50 (73.5)	0.33	35 (71.4)	15 (78.9)
**Major clinical presentation**					
Cognitive decline /behavior changes	NA	11 (16.2)	NA	7 (14.3)	4 (21.1)
Focal neurological deficits	NA	42 (61.8)		31 (63.3)	11 (57.9)
Headache	NA	8 (11.8)		6 (12.2)	2 (10.5)
Transient focal neurological episodes	NA	2 (2.9)		2 (4.1)	0
Unconsciousness	NA	5 (7.4)		3 (6.1)	2 (10.5)
**Past history**					
Hypertension	38 (40.0)	48 (70.6)	<0.001	36 (73.5)	12 (63.2)
Diabetes mellitus	14 (14.7)	15 (22.1)	0.23	11 (22.4)	4 (21.1)
Dyslipidemia	17 (17.9)	7 (10.3)	0.18	7 (14.3)	0
Pre-existing dementia	0	10 (14.7)	<0.001	4 (8.2)	6 (31.6)
Smoking	32 (33.7)	16 (23.5)	0.16	11 (22.4)	5 (26.3)
Antiplatelet drug use	10 (10.5)	21 (30.9)	0.001	16 (32.7)	5 (26.3)
Anticoagulant drug use	1 (1.1)	4 (5.9)	0.16	3 (6.1)	1 (5.3)
**Brain MRI findings**					
Lobar CMBs count	0 (0-0)	13.0 (5-64)	<0.001	22 (5-73)	10 (5-43)
cSS presence	2 (2.1)	26 (38.2)	<0.001	16 (32.7)	10 (52.6)
cSS extent	0 (0-0)	0 (0-1)	<0.001	0 (0-1)	1 (0-2)
WMH Fazekas score	2 (2-3)	4 (4-6)	<0.001	4 (4-5)	4 (4-6)
CSO-PVS	2 (1-2)	2 (2-3)	0.001	2 (2-2)	3 (2-3)
Lacune	0 (0-0)	1 (0-2)	<0.001	1 (0-2)	0 (0-1)
Total MRI burden of SVD	1 (0-2)	3.5 (3-4)	<0.001	3 (3-4)	4 (4-5)
**Blood biomarkers**					
Neurofilament light chain, pg/mL	14.3(10.3-20.4)	70.1 (22.3-320.0)	<0.001	36.6 (19.9-106.0)	368.6 (123.3-790.9)
β-amyloid 40, pg/mL	229.3 ± 61.9	243.3 ± 71.9	0.19	244.9 ± 57.5	239.1 ± 101.9
β-amyloid 42, pg/mL	10.1 ± 2.4	10.2 ± 3.0	0.79	10.5 ± 2.7	9.5 ± 3.6
β-amyloid 42/40	0.047 ± 0.015	0.043 ± 0.006	0.035	0.043 ± 0.007	0.042 ± 0.005
Total tau, pg/mL	2.9 (2.2-4.1)	3.7 (2.6-4.8)	0.011	3.7 (2.6-4.3)	4.4 (2.6-5.7)

Abbreviations: CAA, cerebral amyloid angiopathy; ICH, intracerebral hemorrhage; CMBs, cerebral microbleeds; cSS, cerebral superficial siderosis; WMH, white matter hyperintensities; CSO-PVS, centrum semiovale perivascular space; SVD, small vessel disease.

Data are shown as n (%) for categorical variables, mean ± SD for normally distributed continuous variables or median (interquartile range) for non-normally distributed continuous variables.

**Table 2:** Cox regression models for ICH recurrence after first-ever probable CAA-ICH.

**Variables**	**Univariable analyses**		Multivariable analyses*	
	**HR (95% CI)**	***P* Value**	**HR (95% CI)**	***P* Value**
**Clinical and imaging risk factors for CAA-ICH recurrence**				
Age (per year increase)	1.06 (1.01–1.11)	0.032		
Sex (male vs. female)	0.79 (0.26–2.40)	0.67		
Pre-existing dementia (yes vs. no)	2.19 (0.81–5.90)	0.12		
Total MRI burden of SVD (per point increase)	1.77 (1.21–2.60)	0.003	1.77 (1.21–2.60)	0.003
cSS presence (yes vs. no)	1.77 (0.72–4.39)	0.22		
Disseminated cSS (yes vs. no)	2.15 (0.83–5.56)	0.12		
Lobar CMBs count (per CMB increase)	0.99 (0.98–1.00)	0.17		
WMH Fazekas score (per point increase)	0.98 (0.70–1.38)	0.90		
CSO-PVS (per point increase)	1.85 (1.08–3.18)	0.026		
**Blood biomarkers for CAA-ICH recurrence†**				
Total MRI burden of SVD (per point increase)	1.77 (1.21–2.60)	0.003	2.31 (1.39–3.83)	0.001
Neurofilament light chain	2.03 (1.5 –2.74)	<0.001	2.14 (1.57–2.93)	<0.001
β-amyloid 40	0.23 (0.04–1.41)	0.11		
β-amyloid 42	0.12 (0.02–0.64)	0.013		
β-amyloid 42/40	0.22 (0.01–6.20)	0.38		
Total tau	1.71 (0.71–4.13)	0.24		

Abbreviations: CAA, cerebral amyloid angiopathy; ICH, intracerebral hemorrhage; HR, hazard ratio; SVD, small vessel disease; CMBs, cerebral microbleeds; cSS, cerebral superficial siderosis; WMH, white matter hyperintensities CSO-PVS, centrum semiovale perivascular space.

*The multivariable models included only the covariables for which HRs were provided.

†All variables of blood biomarkers were continuous and Log-transformed.

### Comparisons of blood biomarkers between CAA-ICH cases and controls

Compared to healthy controls, cases with CAA-ICH showed elevated NfL (median [IQR], 14.3 [10.3-20.4] vs. 70.1 [22.3-320.0] pg/ml, p<0.001) and total tau (median [IQR], 2.9 [2.2-4.1] vs. 3.7 [2.6-4.8] pg/ml, p=0.011) levels, and decreased Aβ42/Aβ40 (mean ± SD, 0.047±0.015 vs. 0.043±0.006, p=0.035) levels, with no differences in Aβ42 (mean ± SD, 10.1±2.4 vs. 10.2±3.0 pg/mL, p=0.79) or Aβ40 (mean ± SD, 229.3±61.9 vs. 243.3±71.9 pg/mL, p=0.19) alone (Table 1).

### Clinical model for ICH recurrence prediction in CAA

**Figure 2:**
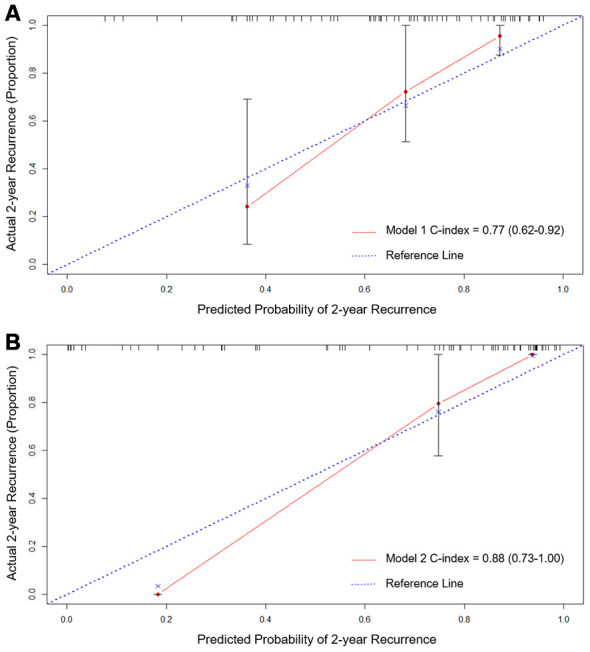
****Calibration curves of Model 1 (**A**) total MRI burden of SVD) and Model 2 (**B**) total MRI burden of SVD and blood NfL) for predicting CAA-ICH recurrence using internal bootstrap validation. Abbreviations: CAA, cerebral amyloid angiopathy; ICH, intracerebral hemorrhage; SVD, small vessel disease; NfL, neurofilament light chain.

All the potential clinical and imaging risk factors for recurrent ICH were assessed in our cohort. Total MRI burden of SVD predicted risk of ICH recurrence best (adjusted hazard ratio [HR] 1.77, 95% confidence intervals [CI] 1.21-2.60, *P*=0.003), none of other clinical or imaging markers contributing with additional predictive value (Table 2). Model 1 of total MRI burden of SVD was generated with Harrell’s concordance index (c-index) of 0.77 (95% CI, 0.62-0.92) (Figure 2A).

### Blood biomarkers for ICH recurrence prediction in CAA

Then, blood biomarkers of Aβ42 and NfL were associated with CAA-ICH recurrence in univariable analyses. Since there were only 19 ICH recurrences in our cohort, a maximum of two predictors were allowed in the multivariable model. Multivariable Cox regression analyses with forward selection demonstrated that blood NfL was associated with CAA-ICH recurrence (adjusted HR 2.14, 95% CI 1.57-2.93) independent of total MRI burden of SVD (adjusted HR 2.31, 95% CI 1.39-3.83) (Table 2). After adding NfL to the clinical model (Model 1), Model 2 of total MRI burden of SVD and NfL showed c-index of 0.88 (95% CI, 0.73-1.00) with improvement compared to Model 1 (*P* =0.019) (Figure 2B). The calibration curve for probability of recurrent ICH from internal bootstrap validation demonstrated satisfactory agreement between actual and predicted probabilities (Figure 2A, 2B).

**Figure 3:**
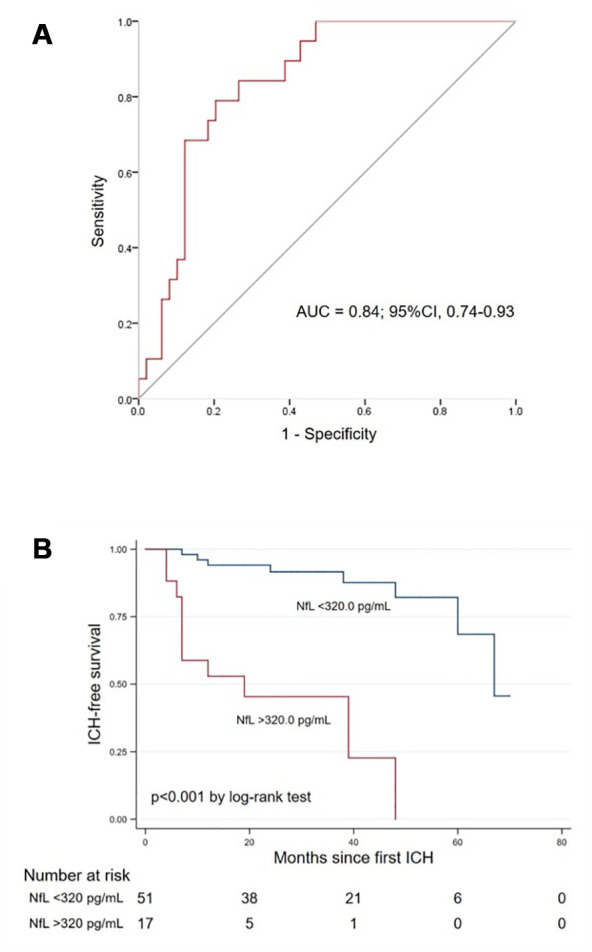
****(**A**) Receiver operating characteristic curve of blood NfL for predicting ICH recurrence in cases with CAA. (**B**) Kaplan–Meier curve showing the ICH-free survival probability in CAA-ICH cases with blood NfL above vs. below 320.0 pg/mL. Abbreviations: NfL, neurofilament light chain; CAA, cerebral amyloid angiopathy; ICH, intracerebral hemorrhage; AUC, area under the curve.

When plotting the receiver operating characteristic (ROC) curve, the area under the curve of blood NfL for predicting ICH recurrence in cases with CAA-ICH was 0.84 (95% CI 0.74-0.93, p<0.001, Figure 3A). In Kaplan–Meier analysis, cases with blood NfL level above the highest tertile (>320.0 pg/mL) had shorter ICH-free survival time than those with NfL below 320.0 pg/mL (log-rank test, *P* <0.001, Figure 3B).

### Association of NfL and ICH volume, baseline NIHSS and clinical outcome

Further, blood NfL level was well correlated with ICH lesion volume (adjusted coefficient 4.71, 95% CI 0.68-8.73), baseline NIH Stroke Scale (NIHSS) score (adjusted odds ratio [OR] 1.68, 95% CI 1.22-2.31) and 6-month modified Rankin scale (mRS) score (adjusted OR 1.75, 95% CI 1.22-2.51) in multivariable analyses (Table 3).

**Table 3:** The association between blood NfL level and ICH volume, baseline NIHSS and clinical outcome.

**Variables**	**Unadjusted**			**Multivariable adjusted**		
	**Coefficient (95% CI)**	***P* Value**	**Coefficient (95% CI)**	***P* Value**	**Coefficient (95% CI)**	***P* Value**
Log NfL and ICH volume	5.95 (2.33–9.56)	0.002	5.76 (2.12–9.41)*	0.002	4.71 (0.68-8.73)^†^	0.023
**Variables**	**OR (95% CI)**	***P* Value**	**OR (95% CI)**	***P* Value**	**OR (95% CI)**	***P* Value**
Log NfL and baseline NIHSS (per point increase)	1.88 (1.37–2.58)	<0.001	1.83 (1.34–2.52)*	<0.001	1.68 (1.22-2.31)^#^	0.002
Log NfL and 6-month mRS (per point increase)	2.28 (1.63–3.18)	<0.001	2.26 (1.61–3.17)*	<0.001	1.75 (1.22-2.51)^†^	0.002

Abbreviation: NfL, neurofilament light chain; ICH, intracerebral hemorrhage; OR, odds ratio; NIHSS, NIH stroke scale; mRS, modified Rankin scale.

*Adjusted for total MRI burden of SVD.

†Adjusted for age, baseline NIHSS and total MRI burden of SVD.

#Adjusted for age, ICH volume and total MRI burden of SVD.

## DISCUSSION

In this study, we established a prospective cohort of cases diagnosed with first probable CAA-ICH in China, and explored SVD imaging markers and blood biomarkers of Aβ42, Aβ40, total tau and NfL for prediction of clinical outcomes in CAA. Blood biomarkers of Aβ42/40, total tau and NfL changed between CAA-ICH and controls. For predicting ICH recurrence in CAA, total MRI burden of SVD and NfL were the best clinical and blood biomarkers respectively. The performance of a model to predict CAA-ICH recurrence using MRI burden of SVD alone increased with the addition of NfL (c-index from 0.77 to 0.88, p=0.019). NfL also correlated to disease severity and clinical outcomes in CAA. We demonstrated that blood NfL assist the prediction of ICH recurrence and clinical outcomes, as well as assessment of clinical severity in CAA.

CAA occupies 14.6% in all subtypes of ICH in Chinese population [15], but to our knowledge no prospective Chinese CAA cohort with ICH recurrence has been reported. The rate of ICH recurrence in our study was 27.9% after a median follow-up of 2.4 years, similar to that of Massachusetts General Hospital (MGH) cohort. Our study found that total MRI burden of SVD other than any single SVD marker, was the only imaging biomarker of recurrent CAA-ICH. This cumulative score exerted gross effects of SVD on brain [16]. Previous studies found that it was independently associated with CAA-related vasculopathic changes and symptomatic CAA-ICH in a neuropathologic cohort and predicted dementia conversion in CAA patients without ICH [16, 17]. It was also related to higher ICH recurrence risk in a large prospective cohort from MGH, but its effect was mainly from the disseminated cSS [2, 18]. Although cSS has been determined as the most important MRI prognostic factor of recurrent ICH in CAA from a meta-analysis [2], there are several different clinical features between all the reported and our cohorts. Different ethnic backgrounds of Asian and White people may lead to various pathophysiological processes for SVD, since Asian patients tend to have a higher prevalence of hypertension and overall SVD load [19, 20]. Patients with CAA in our cohort showed extremely higher CMBs burden but similar cSS severity compared to those in other reported cohorts, which indicated advanced bleeding-prone microangiopathy of our patients. Further, to exclude effects of previous ICH on imaging and blood markers, we only recruited patients with first onset of probable CAA-ICH. These might explain that total MRI burden of SVD instead of cSS was predictive of ICH recurrence in CAA in our cohort.

Emerging data focus on the MRI signatures for CAA, while the utility of blood biomarkers in CAA is still not well validated. NfL was identified to be an independent biomarker of recurrent ICH for CAA in our cohort. It was also related to the baseline disease severity, lesion volume, and clinical outcomes at 6 months. Our findings well correlated with another study of patients with recent small subcortical infarcts, which demonstrated baseline NfL level was associated with infarct size and the occurrence of new lesion at 3 months [21]. Measuring NfL is of great help for the risk stratification in CAA-ICH patients, especially in those who could not take MRI scan in the acute phase of ICH, although the cost is relatively expensive. As a biomarker of the axonal injury, elevated blood NfL levels are observed in almost all neurodegenerative disorders as well as vascular conditions [14]. CAA is a SVD disease intermixed with the neurodegenerative process, but previous studies mostly focused on the pathophysiological process of Aβ-triggered vascular damage [8]. Our findings demonstrate that the neuroaxonal degeneration does prospectively contribute to the development of ICH in CAA, emphasizing the importance of pathologies other than Aβ. The neuroaxonal injury could be a potential therapeutic target for CAA-ICH.

Compared to controls, patients with CAA showed elevated total tau and decreased Aβ42/Aβ40 levels with no differences in the levels of Aβ42 or Aβ40 itself alone. None of Aβ42, Aβ40 or tau was independently predictive of ICH recurrence in CAA. Since all the blood samples of patients with CAA were collected at the acute phase of ICH, it was hard to tell whether CAA or the event of ICH lead to the change of levels. The blood Aβ42/Aβ40 ratio might eliminate the effect of acute stroke event itself. Previous data confirmed that the decreased plasma Aβ42/Aβ40 accurately diagnosed brain amyloidosis in cognitively normal participants [22] and the decreased cerebrospinal fluid (CSF) Aβ42 and Aβ40 (the Aβ42/Aβ40 was not studied) served as biomarkers for diagnosing CAA [6]. Our findings raised the possibility that blood Aβ42/Aβ40 might help distinguish CAA from controls as blood Aβ detected by SIMOA was well correlated with CSF Aβ [23]. Blood taken from CAA-ICH more than 6 months after the hemorrhagic event or from CAA with cognitive decline were needed to confirm its diagnostic value.

Our study has several limitations. First, the hospital-based recruitment from a specific geographical area might limit the generalizability of our results, and the number of patients with probable CAA-ICH in our study was not that large as that of MGH cohort [2]. We designed a prospective cohort to demonstrate the CAA-ICH recurrence rate and relevant predictors in China with 2.4 years’ follow-up. Even in this relatively small sample, we were able to determine the novel blood biomarker to predict recurrent ICH in CAA. Second, we did not collect blood samples from patients with other types of ICH as comparisons, so it was hard to explore the effect of the pathophysiological process of CAA and the acute event of ICH on the levels of blood biomarkers. Third, serial blood samples were not available to determine which timepoint was the best for the prediction of clinical outcomes in CAA, since levels of most inflammatory factors varied over time after onset. For NfL, it increased starting from admission, peaked at day 7 and remained elevated till 6 months post ischemic stroke, with no differences between that of day 1, day 2 and day 3 [24, 25]. We collected blood samples between 1-3 days of ICH onset and acute phase blood biomarkers might be of the greatest value to evaluate disease severity and prognosis.

In conclusion, acute phase blood NfL is a significant biomarker of CAA-ICH recurrence independent of total MRI burden of SVD and is associated with disease severity and clinical outcome of CAA. Thus, NfL is a promising addition to MRI burden of SVD to predict CAA-ICH recurrence.

## MATERIALS AND METHODS

### Study population

We prospectively collected cases with first CAA-ICH admitted at Huashan Hospital Fudan University (Shanghai, China) from January 2014 to September 2018 and at First Affiliated Hospital of University of Science and Technology of China (Hefei, China) from January 2018 to September 2018, and sex- and age-matched healthy elder adults from participants in Shanghai Aging Study during the same period as previously reported [26]. For cases with CAA, the inclusion criteria included: 1) first spontaneous lobar ICH within 48 hours of onset and pathologically or clinically diagnosed with probable CAA according to the modified Boston criteria [27]; 2) cases survived the first 30 days; 3) available blood samples within 24 hours after admission, CT scan within 24-48 hours and MR images including T1, T2, T2 fluid attenuated inversion recovery (FLAIR) and susceptibility weighted imaging (SWI) within 14 days after the event; 4) available follow-up information for mRS at six months and recurrent ICH. We personally inquired about the detailed medical history and examined the patient at baseline. The assessment of pre-existing dementia was done at baseline with the short version of the Informant Questionnaire on Cognitive Decline in the Elderly (IQCODE) [28]. All patients were regularly followed-up every six months by phone calls or face-to-face interview until the ICH recurrence, death or October 2019. ICH recurrence was defined as a new symptomatic lobar ICH event confirmed by corresponding lesion on CT or SWI scan during follow-up period [2]. The current study was approved by local ethics committees. The written informed consent was provided by the patient or a legally responsible relative.

### Blood samples and biomarkers measurements

Blood samples were drawn from the peripheral vein of each participant and collected in tubes with and without EDTA, within 24 hours since admission for patients with CAA and during follow-up period for healthy elder people. After centrifugation at 3,000rpm for 15 minutes, the plasma and serum were kept at −80° C until measurement.

All measures were performed blinded to the clinical data. Serum NfL and plasma Aβ40, Aβ42 and total tau were measured using the SIMOA NF-light assay (Cat No: 102258) and Neurology 3-Plex A (Cat No: 101995) respectively, per manufacturer instructions (Quanterix, MA, USA) on a HD-1 platform at GBIO (Hangzhou, China) with dilution at 1:4 ratio. All samples were duplicated for detection and intra- and inter-assay concentration variabilities were <15%.

### MRI protocols and imaging analysis

CAA patients and healthy controls were scanned on Siemens 3.0T MRI and GE 3.0T scanner separately. Imaging included T1-weighted, T2-weighted, FLAIR and SWI, and parameters of the sequences were shown in Supplementary Materials.

Two experienced raters (Y.W. and Y.X.) blinded to the clinical history independently evaluated SVD imaging markers according to the STRIVE (STandards for ReportIng Vascular changes on nEuroimaging) [29] and validated scales. If there was a disagreement a third rater (Y.S.) was consulted. CMBs which were not immediately adjacent to the lobar ICH were counted on SWI and classified into deep and lobar categories [30]. cSS presence was defined as hypointense curvilinear signal intensity in the superficial layers of the cerebral cortex on SWI, away from at least 2 sulci of the hemorrhagic lesion, and its extent was rated as focal (less than 3 sulci) or disseminated (>3 sulci) distribution on SWI [31]. PVS were measured by a 4-point scale (0 = no PVS, 1 = ≤10 PVS, 2 = 11–20 PVS, 3 = 21–40 PVS, 4 = >40 PVS) in the CSO on T2-weighted images [32] and WMH severity was evaluated using Fazekas scale on FLAIR [33], both from the ICH-free hemisphere in patients with CAA. The total MRI burden of SVD was calculated by accumulating CMBs number, WMH severity, cSS presence and extent and CSO-PVS severity, ranging from 0 to 6 points [16]. ICH volume was measured by 1/2*(length*width*height) on CT scan.

### Statistical analysis

Descriptive statistics included counts with percentages [n(%)] and mean ± SD for normally distributed continuous variables or median with IQR for non-normally distributed continuous variables. We compared demographic, clinical, imaging characteristics and the distribution of blood biomarkers between healthy controls and cases with CAA-ICH using one-way analysis of variance or Mann-Whitney test for continuous variables and χ2 test or Fisher exact test for categorical variables. We log-transformed blood biomarkers to obtain a normal distribution for further analyses of cases with CAA-ICH.

We used univariable and multivariable Cox regression analyses with forward selection to calculate HR with 95% CI and establish the prognostic model for CAA-ICH recurrence. First, we identified clinical and imaging variables associated with CAA-ICH recurrence (p<0.05) in univariable analyses and variables with known potential clinical significance (including age, sex, pre-existing dementia, cSS presence and extent, CSO-PVS severity, lobar CMBs count, WMH severity and total MRI burden of SVD) [1–3, 5] to determine the clinical model (Model 1). Then biomarkers of Aβ40, Aβ42, Aβ42/40, total tau and NfL were added to build the ‘clinical + biomarker’ model (Model 2). The performance of these prognostic models was evaluated by discrimination (c-index) and calibration (internal bootstrap validation) in R software. Comparisons of the models’ c-indexes were made using the compareC package. We explored the ability of the best biomarker in predicting recurrent ICH by a ROC curve and estimates of the area under the curve. We also analyzed the Kaplan–Meier plot with log-rank test of the predictor above and below the upper tertile. Last, we used univariable and multivariable regression models to estimate the association between the blood biomarker and baseline ICH volume, 6-month mRS (with age, baseline NIHSS score and total MRI burden of SVD as covariables), and baseline NIHSS score (with age, ICH volume and total MRI burden of SVD as covariables). Coefficient and OR with 95% CI were reported.

Statistical analyses were performed using STATA version 16.0 (StataCorp, Ltd, College Station, TX) and R software version 3.1.2 (Institute for Statistics and Mathematics, Vienna, VIC, Austria). A two-tailed p<0.05 was considered as significant.

## Supplementary Material

Supplementary Materials
