# Supplementary material for: Neurofilament light chain predicts risk of recurrence in cerebral amyloid angiopathy-related intracerebral hemorrhage
Source: Aging (Albany NY). 2020 Nov 18;12(23):23727–38. doi: 10.18632/aging.103927 (PMC7762473; doi:10.18632/aging.103927)
Supplement: Supplementary Materials [file aging-12-103927-s001.pdf]

SUPPLEMENTARY MATERIALS

MRI parameters

All patients with CAA were scanned on a Siemens MAGNETOM Verio 3.0T MRI system. Parameters of the sequences were as follows: T1 MPRAGE (repetition time [TR]/echo time [TE] 1780/2.9 ms, in-plane matrix 512 × 512, slice thickness 1.0 mm), T2 SPACE (TR/TE 3000/330 ms, in-plane matrix 320 × 320, slice thickness 2.0 mm), T2 FLAIR (TR/TE 9000/99 ms, in-plane matrix 512 × 512, slice thickness 2.0 mm) and SWI (TR/TE 28/20 ms, in-plane matrix 350 × 448, slice thickness 1.5 mm) sequences.

For healthy controls, MRIs were obtained by GE MR750 discovery 3.0T scanner, including T1 BRAVO (TR/TE 8.2/3.2 ms, in-plane matrix 512 × 512, slice thickness 1.0 mm), T2 PROPELLER (TR/TE 10400/80 ms, in-plane matrix 512 × 512, slice thickness 2.0 mm), Cor CUBE FLAIR (TR/TE 6000/90 ms, in-plane matrix 512 × 512, slice thickness 2.0 mm) and SWI (TR/TE 78/45 ms, in-plane matrix 512 × 512, slice thickness 2.0 mm) sequences.
